# Capability of GPT-4V(ision) in the Japanese National Medical Licensing Examination: Evaluation Study

**DOI:** 10.2196/54393

**Published:** 2024-03-12

**Authors:** Takahiro Nakao, Soichiro Miki, Yuta Nakamura, Tomohiro Kikuchi, Yukihiro Nomura, Shouhei Hanaoka, Takeharu Yoshikawa, Osamu Abe

**Affiliations:** 1 Department of Computational Diagnostic Radiology and Preventive Medicine The University of Tokyo Hospital Bunkyo-ku, Tokyo Japan; 2 Department of Radiology School of Medicine Jichi Medical University Shimotsuke, Tochigi Japan; 3 Center for Frontier Medical Engineering Chiba University Inage-ku, Chiba Japan; 4 Department of Radiology The University of Tokyo Hospital Bunkyo-ku, Tokyo Japan

**Keywords:** AI, artificial intelligence, LLM, large language model, language model, language models, ChatGPT, GPT-4, GPT-4V, generative pretrained transformer, image, images, imaging, response, responses, exam, examination, exams, examinations, answer, answers, NLP, natural language processing, chatbot, chatbots, conversational agent, conversational agents, medical education

## Abstract

**Background:**

Previous research applying large language models (LLMs) to medicine was focused on text-based information. Recently, multimodal variants of LLMs acquired the capability of recognizing images.

**Objective:**

We aim to evaluate the image recognition capability of generative pretrained transformer (GPT)-4V, a recent multimodal LLM developed by OpenAI, in the medical field by testing how visual information affects its performance to answer questions in the 117th Japanese National Medical Licensing Examination.

**Methods:**

We focused on 108 questions that had 1 or more images as part of a question and presented GPT-4V with the same questions under two conditions: (1) with both the question text and associated images and (2) with the question text only. We then compared the difference in accuracy between the 2 conditions using the exact McNemar test.

**Results:**

Among the 108 questions with images, GPT-4V’s accuracy was 68% (73/108) when presented with images and 72% (78/108) when presented without images (*P*=.36). For the 2 question categories, clinical and general, the accuracies with and those without images were 71% (70/98) versus 78% (76/98; *P*=.21) and 30% (3/10) versus 20% (2/10; *P*≥.99), respectively.

**Conclusions:**

The additional information from the images did not significantly improve the performance of GPT-4V in the Japanese National Medical Licensing Examination.

## Introduction

The field of natural language processing is rapidly developing with the advent of large language models (LLMs). LLMs are models trained with massive text data sets and achieve the capability to understand and generate text in natural languages. With the introduction of ChatGPT (OpenAI) [1] and other LLM-based chatbot services, many people have started to benefit from the use of LLMs. Although ChatGPT and its underlying model, generative pretrained transformer (GPT) [2,3], were not specifically developed for medical purposes, they possess a considerable amount of medical knowledge. They have achieved good scores in the United States Medical Licensing Examination [4] and are being explored for various applications for clinical and educational purposes [5-7]. GPT can also understand languages other than English. The latest model, GPT-4, has been reported to achieve passing scores in medical licensing examinations in non–English speaking countries such as Japan, China, Poland, and Peru [8-13].

Despite these successes, there is still a significant challenge in applying LLMs to real-world problems with non–text-based information. Radiological, pathological, and many other types of visual information play a crucial role in determining a patient’s management. Very recently, researchers have proposed multimodal variants of LLMs that can handle not only text but various types of input including images [14]. Providing medical images to multimodal LLMs may realize an even higher accuracy in solving medical-related problems. However, in previous studies on the accuracy rate of medical licensing examinations, questions with images were either not mentioned at all or explicitly excluded from the studies. To the best of our knowledge, no study directly evaluated the performance in solving questions with images. Therefore, in this study, we investigated the image recognition capabilities and limitations of GPT-4V [3,15], one of the most potent publicly available multimodal (vision and language) models, in solving medical questions. We focused on the Japanese National Medical Licensing Examination to examine how the visual information affects GPT-4V’s performance.

## Methods

### Overview

From the questions of the 117th Japanese National Medical Licensing Examination, held in February 2023, we focused on those that included images as part of a question. Since some of these questions can be answered correctly without interpreting images, we measured the benefit of adding image information by comparing the accuracy rates of ChatGPT under two different conditions: (1) with both the question text and associated images and (2) with the question text only.

### Data Set Details

**Figure 1:**
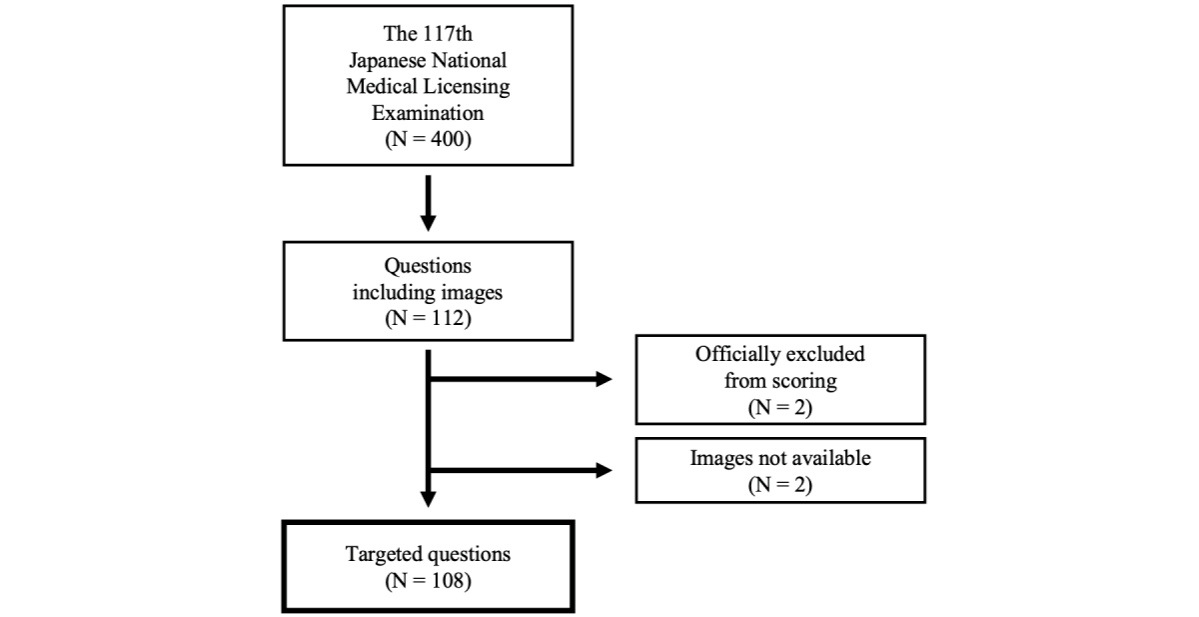
Summary of the questions included in this study.

Figure 1 shows the summary of our data set. The questions and correct answers of the 117th Japanese National Medical Licensing Examination are publicly available for download on the official website of the Ministry of Health, Labour and Welfare [16]. All the questions are in a format in which a specified number of choices, typically 1, are to be selected from 5 options. Of the questions that had images, 2 were officially excluded from scoring because they were either too difficult or inappropriate. Additionally, for 2 questions, images of female genitals were not made public on the aforementioned website. These 4 questions were excluded from our study.

The questions in the Japanese National Medical Licensing Examination were divided into 2 categories: clinical questions and general questions. In clinical questions, clinical information about a specific case is first presented, such as medical history and test results, and answers to questions about the case are required. General questions are about basic medical knowledge, and one is required to choose the correct answer among options for a short question text (typically of 1 or 2 sentences) with an image.

Some clinical questions consisted of multiple subquestions, in which case the background common to all the subquestions was first described, followed by the subquestions. In such cases, each subquestion was individually included in the following analysis if either the subquestion itself or the background part contained an image.

As a result, counting subquestions individually, out of 400 questions, we collected 108 questions that had images, such as photographs of lesions, radiographic images, histopathological images, electrocardiograms, and graphs representing statistical data. Among them, 98 were clinical questions and 10 were general questions.

### Experimental Details

We used ChatGPT (September 25, 2023, version) enabled with GPT-4V, which is a multimodal model capable of processing both text and images. This version of ChatGPT asserts it was trained with information up to January 2022, meaning that it had no direct prior knowledge about our target examination. All the question statements and images were manually entered through ChatGPT’s web interface. One of the authors, TN, who has 10 years of experience as a medical doctor, reviewed the outputs to interpret the response output by ChatGPT.

A new chat session was created for each question and each condition (ie, with or without images). For questions that comprised multiple subquestions, the background information part and each subquestion were entered into ChatGPT in this order within the same chat session. Subquestions without images were also input to provide ChatGPT with enough context, but they were excluded from the accuracy calculations and the subsequent statistical analysis described below.

The questions were presented to ChatGPT without any preceding or custom instructions. Sometimes, ChatGPT did not respond with the specified number of choices, in which case an additional instruction, such as “select only one option“ or “select two options,” was provided in Japanese. This additional instruction produced the correct number of options for all the questions.

### Statistical Analysis

The difference in ChatGPT’s performance between the 2 conditions (ie, with or without images) was analyzed using the exact McNemar test. A *P* value of less than .05 was considered statistically significant. The analysis was conducted using R (version 4.3.1; R Foundation for Statistical Computing).

### Ethical Considerations

This study was conducted solely using publicly available resources, therefore, approval from the institutional review board of our institution was not required.

## Results

Table 1 shows the results of our experiment. ChatGPT correctly answered 68% (73/108) of image-based questions when provided with both the question text and images, whereas it correctly answered 72% (78/108) of image-based questions when only the question text was provided. There was no significant difference in accuracy between these 2 conditions (*P*=.36). For the clinical questions, the accuracies when presented with and without images were 71% (70/98) and 78% (76/98), respectively. For the general questions, the accuracies were 30% (3/10) when presented with images and 20% (2/10) without images. We have included examples of the input and output along with their English translations in Multimedia Appendix 1, and we have also provided a summary of image interpretation for each question where the results differed depending on the presence of image input (N=7+12) in Multimedia Appendix 2.

**Table 1:** Performance of ChatGPT in answering questions from the 117th Japanese National Medical Licensing Examination, when presented with or without associated images for each question.

			With images		
			Correct	Incorrect	Total
**Overall (*P*=.36)**					
	**Without images, n (%)**				
		Correct	66 (61)	12 (11)	78 (72)
		Incorrect	7 (6)	23 (21)	30 (28)
		Total	73 (68)	35 (32)	108 (100)
**Clinical (*P*=.21)**					
	**Without images** **, n (%)**				
		Correct	65 (66)	11 (11)	76 (78)
		Incorrect	5 (5)	17 (17)	22 (22)
		Total	70 (71)	28 (29)	98 (100)
**General (*P*≥.99)**					
	**Without images, n (%)**				
		Correct	1 (10)	1 (10)	2 (20)
		Incorrect	2 (20)	6 (60)	8 (80)
		Total	3 (30)	7 (70)	10 (100)

## Discussion

### Principal Results

In this study, we examined the image recognition capabilities of GPT-4V using questions associated with images from the Japanese National Medical Licensing Examination. To the best of our knowledge, this is the first study in which the capability of multimodal LLM for the Japanese National Medical Licensing Examination was investigated. Contrary to our initial expectations, the inclusion of image information did not result in any improvement in accuracy. Instead, we even observed a slight decrease, albeit not significant. This indicates that, at the moment, GPT-4V cannot effectively interpret images related to medicine. The passing score rate for the 117th Japanese National Medical Licensing Examination is approximately 75% (and 80% for some questions marked as “essential”) [16]. In this study, GPT-4V failed to reach this passing score rate for the questions it was tested on. Considering that 92% of human candidates passed, it implies that the image interpretation skills of GPT-4V will fall short of those possessed by many medical students.

For the clinical questions, in which sufficient clinical information including patient history was available in the text form, GPT-4V was able to choose the correct answers solely from the textual information in the majority (76/98, 78%) of questions, but the addition of images did not improve the accuracy. On the other hand, for the general questions, there was little information in the question text, and GPT-4V had to determine the correct answer by interpreting the images. For these, GPT-4V yielded an accuracy rate that was hardly any better than random guessing even when presented with images. Our results suggest that, for both categories of questions, GPT-4V failed to use visual information to improve its accuracy. We observed that GPT-4V often either explicitly stated that it was unable to interpret the images or failed to provide information beyond what was evident from the question text. In our retrospective review, even in questions where GPT-4V gave correct answers only when presented with images, there were only 2 out of 7 questions where it provided a correct interpretation of the image and used that as a critical clue. Conversely, in questions where GPT-4V provided incorrect answers only when presented with images, it sometimes made incorrect or insufficient interpretations of the images, leading to incorrect answers (4 out of 12).

ChatGPT may serve as a valuable teaching assistant in medical education; however, the inaccuracies in its responses are a significant concern [5,7]. Our current findings suggest that, especially with medical-related images, GPT-4V should not be relied upon as a primary source of information for medical education or practice. If used, extreme caution should be exercised regarding the accuracy of its responses. OpenAI officially states [15] that they “do not consider the current version of GPT-4V to be fit for performing any medical function or substituting professional medical advice, diagnosis, or treatment, or judgment” due to its imperfect performance in the medical domain. Yang et al [17] have comprehensively examined the capabilities of GPT-4V in various tasks including medical image understanding and radiology report generation, and they stated that GPT-4V could correctly diagnose some medical images. However, as they acknowledge, their results contained a considerable number of errors, such as overlooking obvious lesions and errors in laterality. According to the case studies by Wu et al [18], GPT-4V could recognize the modality and anatomy of medical images, but it could hardly make accurate diagnoses and its prediction relied heavily on the patient’s medical history. The results of our experiment supported these previous reports.

Considering the well-known high performance of GPT-4V in more generic image recognition tasks [3,17], the most probable reason for its limited image recognition performance in the medical field is that it may simply not have been trained with a sufficient number of medical-related images. LLMs are trained with a vast data set available on the internet, but medical images are not as readily accessible, partly due to privacy concerns. Some researchers are now working on developing multimodal LLMs specialized for medicine based on open-source LLMs [19,20]. These models use publicly available data sets that combine medical images and text, including MIMIC-CXR [21], which contains chest x-ray images with their associated reports, and PMC-OA [22], a compilation of the figures and captions from open-access medical journal papers. The rise of multimodal LLMs is expected to stimulate the publication of more such data sets, thereby advancing the development of multimodal LLMs in the medical field. Moreover, although there are limited medical-related images publicly available on the internet, hospitals have a vast amount of image data. A large part of this is accompanied by textual interpretations in the form of reports or medical records, which may serve as an ideal data set for training multimodal LLMs. In highly specialized domains such as medicine, there remains a significant value in developing domain-specific models using such medical data sets.

### Limitations

This study had several limitations. First, ChatGPT was not provided any prior instructions and was directly presented with only the questions themselves. This might have negatively affected its capability to interpret images as the capabilities of LLMs are known to be affected by such “prompt engineering.” This will be a subject for future investigation. Second, this study specifically targeted the Japanese National Medical Licensing Examination, and thus, further analysis is necessary to determine whether its conclusions can be generalized to questions in other languages or of different types. However, as mentioned earlier, the limited capability of GPT-4V to interpret medical images has also been demonstrated in other studies focusing on English [17,18], and our results are consistent with those findings. Since ChatGPT’s proficiency in non-English interpretation is known to be inferior to that in English interpretation, translating the question text into English before inputting it to ChatGPT might have improved the model’s image interpretation capability. However, in a previous study by Yanagita et al [10], in which nonimage questions from the Japanese National Medical Licensing Examination were the target, satisfactory results were achieved even when the questions were input in Japanese. Thus, we adopted the same approach in our study. Third, although our results were based on the same version of ChatGPT and the same question was evaluated with and without images on the same day, we cannot exclude the possibility that different models were used internally. Lastly, only a single evaluation was conducted for each condition and question. ChatGPT’s outputs have some randomness, and responses may differ across multiple evaluations. With ChatGPT’s application programming interface, users can programmatically control the degree of randomness by specifying a parameter called *temperature* and obtain mostly deterministic responses. However, during the time of this study, the application programming interface for GPT-4V was not available.

### Conclusions

At present, GPT-4V’s capability to interpret medical images may be insufficient. In highly specialized fields such as medicine, it is considered meaningful to develop field-specific multimodal models.

## Appendix

Multimedia Appendix 1 Examples of inputs and outputs from GPT-4V.

Multimedia Appendix 2 Summary of image interpretation by GPT-4V.

## Abbreviations

GPT: generative pretrained transformer
LLM: large language model
