# Supplementary material for: Capability of GPT-4V(ision) in the Japanese National Medical Licensing Examination: Evaluation Study
Source: JMIR Med Educ. 2024 Mar 12;10:e54393. doi: 10.2196/54393 (PMC10966435; doi:10.2196/54393)
Supplement: Multimedia Appendix 1 [file mededu_v10i1e54393_app1.pdf]

Q&A Examples

Contents

Contents 1
Introduction 2
English Translation 2
Example 1 (Question E7, General) 2
Question 2
Correct Image Diagnosis 2
Correct Answer 2
GPT-4V Response without Image (Incorrect) 3
GPT-4V Response with Image (Correct) 3
Example 2 (Question A26, Clinical) 4
Question 4
Correct Image Diagnosis 5
Correct Answer 5
GPT-4V Response without Image (Correct) 5
GPT-4V Response with Image (Incorrect) 6
Original Japanese 7
Example 1 (Question E7, General) 7
Question 7
Correct Image Diagnosis 7
Correct Answer 7
GPT-4V Response without Images (Incorrect) 7
GPT-4V Response with Images (Correct) 8
Example 2 (Question A26, Clinical) 9
Question 9
Correct Image Diagnosis 10
Correct Answer 10
GPT-4V Response without Images (Correct) 10
GPT-4V Response with Images (Incorrect) 11

Introduction

In this document, we showcase actual examples of inputs and outputs from GPT-4V, accompanied by their English translations. The question texts and images are all cited from [the 117th Japanese National Medical Licensing Examination](), and all translations have been performed by the authors.

Sections where GPT-4V accurately interpreted the images are highlighted in blue.
Conversely, sections where its interpretations were incorrect are highlighted in red.

English Translation

Example 1 (Question E7, General)

Question

The electrocardiogram waveform of a patient who has fainted is shown separately (Supplement No. 2). Which of the following should be administered immediately?
a Atropine
b Adrenaline
c Diazepam
d Verapamil
e Lidocaine

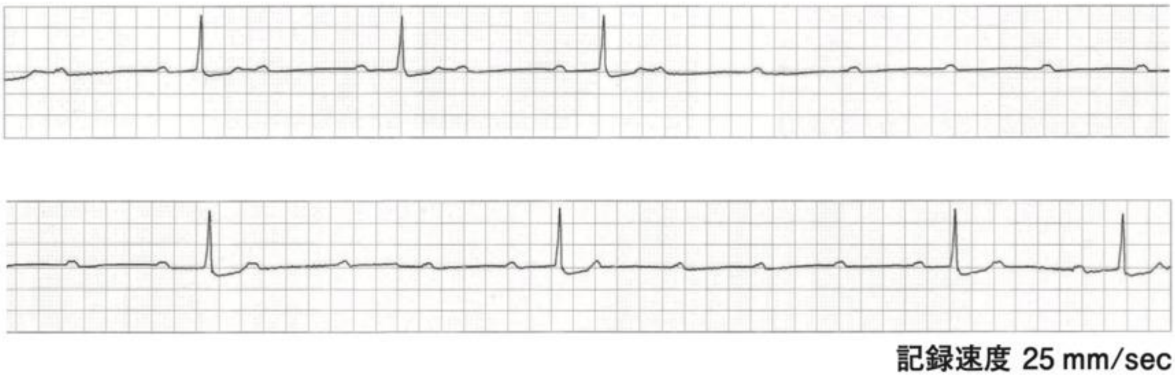

Correct Image Diagnosis

High-grade atrioventricular block

Correct Answer

a

GPT-4V Response without Image (Incorrect)

Unfortunately, as a text-based interface, I do not have the capability to display images or waveforms such as "Supplement No. 2." Therefore, I cannot refer to the specific electrocardiogram waveform.

However, it is possible to infer the clinical situation from the provided options. Based on the electrocardiogram waveform of a patient who has fainted and the response to the listed drugs, I consider the following:

a Atropine: Used for bradycardia or some types of AV block.
b Adrenaline: Used for cardiac arrest or severe hypotension.
c Diazepam: Used for seizures or anxiety.
d Verapamil: Used for certain types of supraventricular tachycardias.
e Lidocaine: Used for bradyarrhythmic or ventricular arrhythmias.

Since the causes of fainting can vary widely, it is not possible to make an accurate choice without seeing the specific electrocardiogram waveform. If you can provide more detailed information about the electrocardiogram waveform or symptoms, I can offer more specific advice.

[Select only one option.]

Understood.

Considering the situation of a patient who has fainted, and taking into account emergencies like cardiac arrest or severe hypotension,
b Adrenaline
would be the most commonly considered option.

However, to provide an accurate answer, details of the electrocardiogram waveform and the patient's clinical condition are necessary.

GPT-4V Response with Image (Correct)

This electrocardiogram waveform may suggest the presence of a third-degree atrioventricular block (complete heart block). In cases of third-degree AV block, where the heartbeat is slow, atropine may be used.

Therefore, the medication that should be administered immediately is:
a Atropine

However, the interpretation of the electrocardiogram and the choice of treatment should be made by a specialist according to the clinical situation. This information is for educational purposes and should not be used as medical advice.

Example 2 (Question A26, Clinical)

Question

A 32-year-old woman presented with complaints of fever, dizziness, and general fatigue. She had been experiencing a low-grade fever of around 37°C for the past six months, accompanied by general fatigue and dizziness. Despite taking over-the-counter cold medicine, her symptoms did not improve, prompting her visit. Her past medical and family histories were unremarkable. Blood pressure measurements showed 120/62 mmHg in the right arm and 80/40 mmHg in the left arm. Vascular bruit was audible from the neck to the left supraclavicular fossa. A Levine 2/6 diastolic murmur was heard at the left sternal border at the level of the third rib. No abnormalities were noted in respiratory sounds. Erythrocyte sedimentation rate was 85 mm/hour. Blood test results: Red blood cells 3.6 million, Hb 10.8 g/dL, white blood cells 9,600 (band neutrophils 10%, segmented neutrophils 70%, eosinophils 1%, basophils 1%, monocytes 4%, lymphocytes 14%). No abnormalities were found in the electrocardiogram and chest X-ray. A separate three-dimensional contrast-enhanced CT scan is provided (Supplement No. 7).
What is the most likely diagnosis?
a. Coarctation of the aorta
b. Sarcoidosis
c. Aneurysm of the aortic arch
d. Stanford Type A aortic dissection
e. Takayasu arteritis

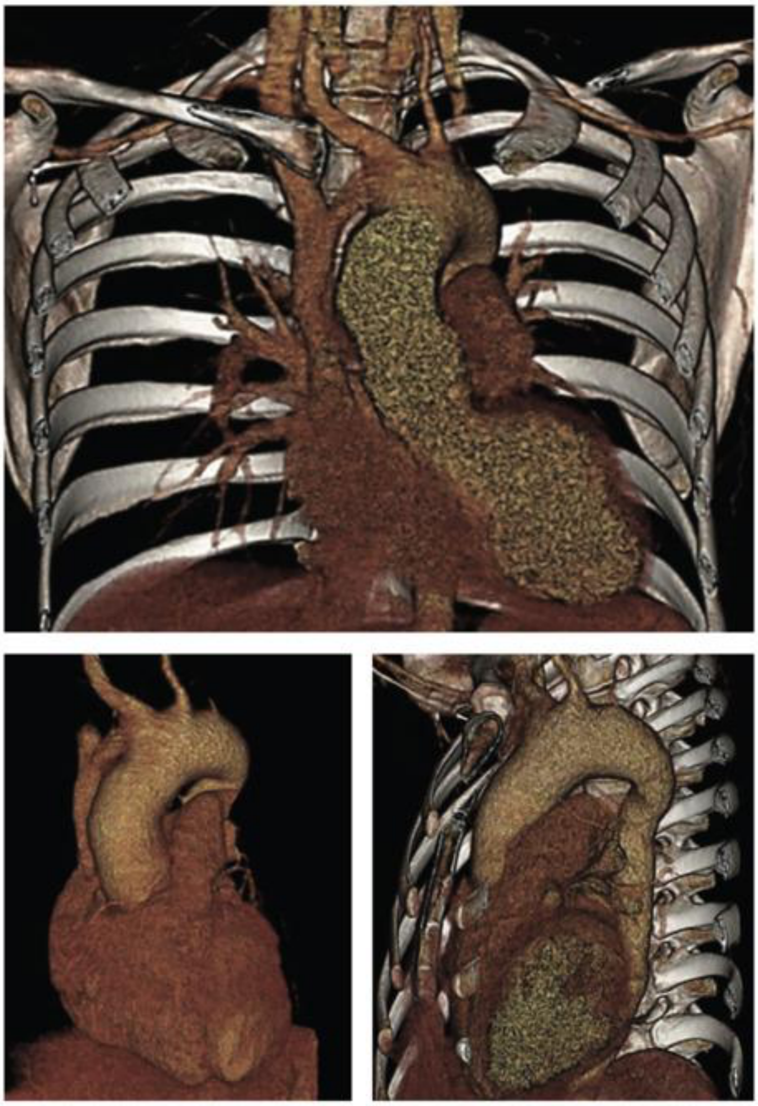

Correct Image Diagnosis

Left subclavian artery occlusion

Correct Answer

e

GPT-4V Response without Image (Correct)

Based on the symptoms and clinical findings of this patient, the following points are noteworthy:

1. The difference in blood pressure between the right and left upper limbs.
2. Vascular bruit from the neck to the left supraclavicular fossa.
3. Diastolic murmur strongest at the third rib along the left sternal border.
4. Elevated erythrocyte sedimentation rate.

These symptoms and findings are likely related to a lesion of the aorta.

Considering the following options:

a. Coarctation of the aorta: Symptoms such as the difference in blood pressure and bruit match this condition, but it is more common in adults with valvular diseases, especially bicuspid valve disease, and is rare in young women.

b. Sarcoidosis: Elevated erythrocyte sedimentation rate and systemic symptoms match, but vascular bruit, cardiac murmur, and the difference in blood pressure do not fit.

c. Aneurysm of the aortic arch: Aneurysms of the aorta can cause bruits, but they are not directly related to the decrease in blood pressure in the left upper limb or diastolic murmur.

d. Stanford Type A aortic dissection: This condition often presents with acute symptoms and does not align with the prolonged low-grade fever and systemic symptoms for six months.

e. Takayasu arteritis: This condition is more common in young women and known to present symptoms such as elevated erythrocyte sedimentation rate, low-grade fever, systemic symptoms, difference in blood pressure, and vascular bruit.

From the above considerations, the most likely diagnosis is:

e. Takayasu arteritis.

GPT-4V Response with Image (Incorrect)

From these images, it seems that images from a three-dimensional contrast-enhanced CT scan are presented. Taking into account the patient's clinical information and the images, we can suggest the most likely diagnosis.

Firstly, the images show abnormalities in the aorta. In particular, there is dilation and deformation in parts of the aorta.

From the patient's clinical information:

Vascular bruit from the neck to the left supraclavicular fossa
Levine 2/6 diastolic murmur strongest at the left sternal border at the third rib
Combining this information, the following diagnoses can be considered:

a. Coarctation of the aorta: While these symptoms could be associated with a narrowing of the aorta, the images do not show clear signs of constriction.
b. Sarcoidosis: This is primarily a lung-affecting disease, and specific signs of this condition are not indicated in this information.
c. Aneurysm of the aortic arch: Considering the abnormal areas in the images and clinical symptoms, this diagnosis is possible.
d. Stanford Type A aortic dissection: Signs of aortic dissection are also suggested in the images, but the clinical signs and symptoms do not fully align.
e. Takayasu arteritis: This disease involves inflammation of the aorta, but the symptoms and image information alone make a definitive diagnosis challenging.

The most likely diagnosis seems to be c. Aneurysm of the aortic arch, but the actual diagnosis should be based on a professional opinion from a physician.

Original Japanese

Example 1 (Question E7, General)

Question

失神している患者の心電図モニターの波形（別冊 No. 2）を別に示す。

直ちに投与すべきなのはどれか。

a　アトロピン

b　アドレナリン

c　ジアゼパム

d　ベラパミル

e　リドカイン

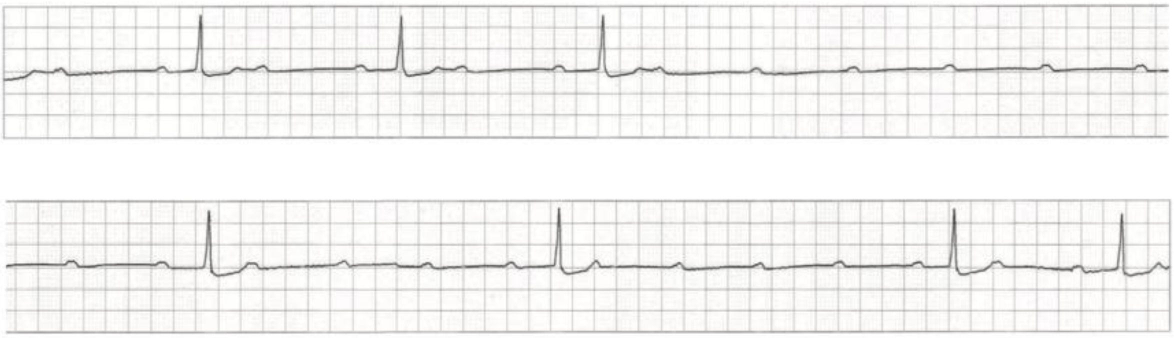

記録速度 25 mm/sec

Correct Image Diagnosis

高度房室ブロック

Correct Answer

a

GPT-4V Response without Images (Incorrect)

残念ながら、テキストベースのインターフェースである私には「別冊 No. 2」という画像や波形を表示する機能はありません。そのため、具体的な心電図の波形を参照することができません。

しかし、提供された選択肢からクリニカルな状況を推測することはできます。失神している患者の心電図モニターの波形と関連する薬物の反応に基づいて以下のように考えます。

a　アトロピン: 低心拍数や一部の AV ブロックに使われる。

b　アドレナリン: 心停止や重度の低血圧に使われる。

c　ジアゼパム: 痙攣や不安に使われる。

d　ベラパミル: 高心拍数の特定の心室上性不整脈に使われる。

e　リドカイン: 徐脈性不整脈や心室性不整脈に使われる。

失神の原因は多岐にわたるため、具体的な心電図の波形を見ないと正確な選択はできません。心電図の波形や症状に関する詳しい情報を提供していただければ、より具体的な助言をすることができます。

[ひとつだけ選択肢を選んでください。]

了解しました。

失神している患者の状況を考えると、心停止や重度の低血圧などの緊急事態を考慮すると、
b　アドレナリン
が最も一般的に考慮される選択肢となります。

しかし、正確な答えを出すためには心電図の波形や患者の臨床状態の詳細が必要です。

GPT-4V Response with Images (Correct)

この心電図モニターの波形は、第三度房室ブロック（完全房室ブロック）を示唆している可能性があります。第三度房室ブロックには、心拍が遅くなるため、アトロピンが使用されることがあります。

したがって、直ちに投与すべきは：
a　アトロピン

しかし、心電図の解釈と治療の選択は、臨床的状況に応じて専門家によって行われるべきです。この情報は教育的な目的のためのものであり、医学的アドバイスとして使用すべきではありません。

Example 2 (Question A26, Clinical)

Question

32 歳の女性。発熱、めまい、全身倦怠感を主訴に来院した。半年前から 37 ℃前後の微熱が持続し、全身倦怠感やめまいも生じてきた。総合感冒薬を内服したが、症状が改善しないため受診した。既往歴と家族歴に特記すべきことはない。血圧は右上肢 120/62 mmHg、左上肢 80/40 mmHg。頸部から左鎖骨上窩にかけて血管雑音を聴取する。心音は胸骨左縁第 3 肋間を最強点とする Levine 2/6 の拡張期雑音を聴取する。呼吸音に異常を認めない。赤沈 85 mm/ 1 時間。血液所見：赤血球 360 万、Hb 10.8 g/dL、白血球 9,600（桿状核好中球 10 %、分葉核好中球 70 %、好酸球 1 %、好塩基球 1 %、単球 4 %、リンパ球 14 %）。心電図と胸部エックス写真に異常を認めない。三次元造影 CT（別冊 No. 7）を別に示す。

最も考えられるのはどれか。

a　大動脈縮窄症

b　サルコイドーシス

c　大動脈弓部大動脈瘤

d　Stanford A 型大動脈解離

e　高安動脈炎〈大動脈炎症候群〉

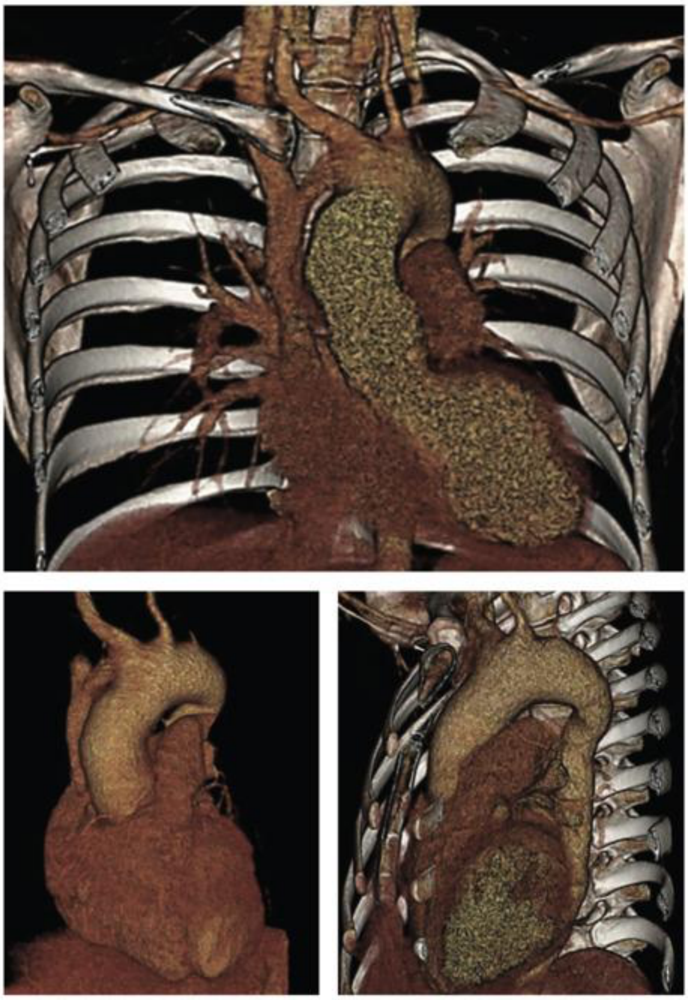

Correct Image Diagnosis

左鎖骨下動脈閉塞

Correct Answer

e

GPT-4V Response without Images (Correct)

この患者の症状と臨床所見から考えると、以下の点が注目されます。

1. 右上肢と左上肢の血圧の差。
2. 頸部から左鎖骨上窩にかけての血管雑音。
3. 胸骨左縁第 3 肋間を最強点とする拡張期雑音。
4. 赤沈の上昇。

これらの症状や所見は、大動脈の病変に関連している可能性が高いです。

以下の選択肢を考慮します：

a 大動脈縮窄症：血圧の差や雑音などの症状はこの病態に合致するが、成人に多いのは弁の病夢、特に二尖弁の病夢であり、若い女性では珍しい。

b サルコイドーシス：赤沈の上昇や全身症状に合致するが、血管雑音や心雑音、血圧の差などの症状は該当しない。

c 大動脈弓部大動脈瘤：大動脈の瘤は雑音の原因となることがあるが、特に左上肢の血圧低下や拡張期雑音とは直接関連しない。

d Stanford A 型大動脈解離：この病夢は急性の症状を呈することが多く、半年もの微熱や全身症状には合致しない。

e 高安動脈炎〈大動脈炎症候群〉：この病態は若い女性に多く、赤沈の上昇、微熱、全身症状、血圧の差、血管雑音などの症状が見られることが知られています。

以上の考察から、最も考えられるのは

e 高安動脈炎〈大動脈炎症候群〉

です。

GPT-4V Response with Images (Incorrect)

これらの画像から、おそらく三次元造影 CT の画像が示されています。患者の臨床的な情報と画像を考慮に入れると、最も考えられる診断を提案できます。

まず、画像には大動脈の異常が示されています。特に大動脈の一部に拡張や変形が見られます。

患者の臨床的情報から：
- 頸部から左鎖骨上窩にかけての血管雑音
- 胸骨左縁第 3 肋間を最強点とする Levine 2/6 の拡張期雑音

これらの情報を組み合わせると、以下の診断が考えられます。

a 大動脈縮窄症: この症状は大動脈の狭窄に関連している可能性がありますが、画像には狭窄の明確な兆候は示されていません。
b サルコイドーシス: 主に肺に影響を及ぼす病気であり、この病状の特有の徴候はこの情報からは示されていません。
c 大動脈弓部大動脈瘤: 画像の異常部位と臨床症状から、この診断が考えられます。
d Stanford A 型大動脈解離: 大動脈解離の兆候も画像で示唆されていますが、臨床的な徴候や症状は完全に一致するわけではありません。
e 高安動脈炎〈大動脈炎症候群〉: この病気は大動脈の炎症を伴いますが、症状や画像の情報だけでは確定的な診断は難しいです。

最も考えられる診断として、c 大動脈弓部大動脈瘤 が妥当であると思われます。ただし、実際の診断は医師の専門的な意見に基づくべきです。
