# Supplementary material for: Capability of GPT-4V(ision) in the Japanese National Medical Licensing Examination: Evaluation Study
Source: JMIR Med Educ. 2024 Mar 12;10:e54393. doi: 10.2196/54393 (PMC10966435; doi:10.2196/54393)
Supplement: Multimedia Appendix 2 [file mededu_v10i1e54393_app2.doc]

**Table S1.** Summary of image interpretation by GPT-4V (incorrect answers without images, correct answers with images).

| **Question No.** | **Image Type** | **Region** | **Correct Image Diagnosis** | ***GPT-4V Image Interpretation** | **Details** |
| --- | --- | --- | --- | --- | --- |
| **A17** | US | Fetus | Twin-to-twin transfusion syndrome | Failed |  |
| **A50** | Photo, CT | Ear | Cholesteatoma | Failed |  |
| **B10** | Graph | Statistics | Lower back pain | Correct? | Provided only the answer without a detailed interpretation of the image |
| **C59** | US | Fetus | Placenta previa | Failed |  |
| **D34** | X-ray, CT | Brain | Aneurysm coiling | Partially Correct | Identified an intracranial device, but was unable to differentiate between a coil and a clip |
| **D70** | MRI, CT | Female Pelvis | Ovarian cancer | Failed |  |
| **E7** | ECG | Heart | High-grade atrioventricular block | Partially Correct | Identified atrioventricular block, but with incorrect degree |

* Indicates if the image interpretation by GPT-4V was correct when presented with both question text and image(s). "Failed" refers to cases where GPT-4V could not perform informative image interpretation. This category includes situations where GPT-4V either explicitly stated it was unable to interpret the image(s) or failed to provide information beyond what was evident from the question text (for example, merely stating 'ultrasound findings are provided').

**Table S2.** Summary of image interpretation by GPT-4V (correct answers without images, incorrect answers with images).

| **Question No.** | **Image Type** | **Region** | **Correct Image Diagnosis** | **GPT-4V Image Interpretation*** | **Details** |
| --- | --- | --- | --- | --- | --- |
| **A26** | CT | Chest | Left subclavian artery occlusion | Incorrect | Incorrectly identified as having aortic dilation |
| **A31** | Photo | Skin | Vitiligo vulgaris | Partially Correct | Correctly identified leukoderma, but failed to correlate it with medical history, leading to an incorrect diagnosis |
| **A39** | Photo, Histopathology | Skin | Melanocytic nevus | Failed |  |
| **A70** | Photo | Equipment | Pipette for stable microbubble test | Partially Correct | Correctly recognizes the pipette, but failed to identify its use in the specific test, resulting in an incorrect conclusion |
| **A74** | CT | Abdomen | Incarcerated inguinal hernia | Failed |  |
| **C8** | US | Heart | Mitral valve | Failed |  |
| **D17** | MRI | Female Pelvis | Ovarian endometrioma | Failed |  |
| **D31** | Histopathology | Kidney | Crescentic glomerulonephritis | Failed |  |
| **D62** | MRI | Brain | Glioblastoma | Correct | Correctly detect and locate the tumor, but chose incorrect options |
| **F57** | Textual | Lab Results | Orthostatic proteinuria | Failed |  |
| **F59** | ECG | Heart | Pulseless electrical activity | Incorrect | Misdiagnosed as ventricular fibrillation |
| **F68** | Nuclear | Brain, Heart | Parkinson disease | Correct | Correctly identified the image findings in DaT scan and MIBG cardiac scan, but chose incorrect option |

* Indicates if the image interpretation by GPT-4V was correct when presented with both question text and image(s). "Failed" refers to cases where GPT-4V could not perform informative image interpretation. This category includes situations where GPT-4V either explicitly stated it was unable to interpret the image(s) or failed to provide information beyond what was evident from the question text (for example, merely stating 'ultrasound findings are provided').
